# Editorial: Enhancement of photosynthesis through light utilization in plants and crops

**DOI:** 10.3389/fpls.2025.1569835

**Published:** 2025-05-19

**Authors:** Sina Fallah, Meijian Yang, Jose R. Peralta-Videa

**Affiliations:** ^1^ Department of Agronomy, Faculty of Agriculture, Shahrekord University, Shahrekord, Iran; ^2^ Center for Climate Systems Research (CCSR), Columbia Climate School, Columbia University, New York, NY, United States; ^3^ Department of Chemistry and Biochemistry, Chemistry and Computer Science Building, The University of Texas at El Paso, El Paso, TX, United States

**Keywords:** photosynthesis, light intensity (irradiance), daily light integral (dli), light spectrum, monochromatic light

The essential role of light in photosynthesis was discovered nearly two and a half centuries ago by the Dutch physician Jan Ingen-Housz, who demonstrated that plants exposed to light restore oxygen (Stirbet et al., 2020). In 1872, Jean Senebier et al. demonstrated that CO_2_ was needed to restore O_2_ in light-exposed plants (Shevela et al., 2019). Since then, thousands of articles have been written describing the role of light in photosynthesis.

Crop production is reduced due to changes in photosynthesis, which is affected by environmental conditions, such as CO_2_ excess/depletion or water shortage. He and Matthews (2023) mentioned that the elevated CO_2_ concentration impacted photosynthesis and reduced yield in soybeans. Water and fertilizer supply also affect photosynthesis and yield. Chastain et al. (2014) evaluated the effects of water deficit on net photosynthesis and lint production in a two-year field experiment with three cotton (*Gossypium hirsutum*) cultivars. The cultivars were ground in a dryland with only rainfall or well-watered during the growing season. The authors found that under water deficit, there was a decrease in stomatal conductance and an increase in photorespiration, resulting in a reduction of net photosynthesis and lint yield (in only one of the seasons).

Cultivation in a controlled environment can help combat climate uncertainties and maintain food supplies in regions with limited arable land. However, this requires a specific structure to improve and maintain photosynthesis (Niu and Masabni, 2018). This Research Topic contains 10 articles discussing key factors impacting photosynthesis and crop production under controlled conditions. Six of these articles discussed different effects of the light spectrum, mainly red and far-red light effects. One presented the impact of cell size on photosynthesis. Another examined the interaction of CO_2_ with light, and the others discussed different aspects of photosynthesis and plant growth, such as light intensity. Light intensity affects photosynthesis in different manners depending on the type of plant. There has been inconsistency in the number of days that plants can tolerate low daily light integral (DLI) days after exposure to a high DLI day of natural light. Previous reports referred to a single day, which practically eliminates the use of supplemental light. Mayorga-Gomez et al. experimented with lettuce (*Lactuca sativa*) plants exposed to a high DLI day (22.5 mol/m^2^*day) followed by a varying number of low DLI days. They reported that lettuce plants exposed to one day of high DLI can endure multiple days of low DLI, which may result in reduced energy consumption. CO_2_ concentration and light intensity affect photosynthesis, stomatal conductance, and leaf transpiration. Lv et al. reported that increased photosynthetically active radiation in rice rapidly increases stomatal conductance, transpiration, and net photosynthesis. Conversely, increasing the CO_2_ concentration gradually decreases stomatal conductance and transpiration, but photosynthesis increases linearly and slowly as leaf development increases until stabilization. However, CO_2_ absorption by leaves depends on several factors, including variations in light and CO_2_ volume. Ogolla Egesa et al. reported that cell size is another factor influencing the photosynthetic rate. They studied Mesoamerican and Andean common beans and found that the former has smaller epidermal cells with higher stomatal density, which allows higher water and CO_2_ conductance. This helps the plants to increase chlorophyll and protein content.

Another light parameter to consider is the spectrum. Changes in the red and far-red spectra have been shown to affect plant development differently. Bi et al. evaluated the effects of changes in the ratio of red to far-red light on biochemical parameters and the nutritional quality of lettuce. They compared the effects of 450 nm blue light + 650 nm red light (control) with 650 nm red light + 730 nm far-red light in a 3:2 ratio (F3). They found that plants exposed to F3 had significantly higher net photosynthetic rate, stomatal conductance, leaf area, aboveground fresh weight, vitamin C, and total soluble sugars. The duration of far-red light, which affects phytochrome, impacts plant development. Igarashi et al. illuminated leaves of arugula for 120–300 sec with LED light of 735 nm (far-red) and 635 nm (red) plus a laser light of 852 nm to produce biospeckles for rapid evaluation of far-red influence. They found that brief exposure to far-red light increased internal activity compared with prolonged exposure. They also found that the response of one-month-old leaves was better than that of three-month-old leaves. Chen et al. reported that reducing red light in full-spectrum LEDs has a significant impact on the growth of the propagation remains of strawberries. White LEDs increased the total dry mass of runner plants by 83%compared to red and blue LEDs. On the other hand, Ke et al. cultivated Micro-Tom and Rejina tomatoes exposed to monochromatic red light, a red/blue light ratio = 3, and white light at 300 µmol/m^2^*s. The monochromatic red light photosynthetic rate resulted in the lowest radiation use efficiency. The highest proportion of blue light (up to 25%) resulted in the highest photosynthetic rate and radiation use efficiency. Blue light produced the best effects on fruit. Similar results were reported by Almeida Lima et al. on microtomato plants exposed for 12 h to blue light at 300 µmol/m^2^*s and 3.7 W/m^2^ UV-B for 1 h daily. These plants had the highest photosynthetic rates and fruits with the highest rutin content compared to red and white light. Lv et al. stated that crop production may increase under suboptimal conditions by improving far-red utilization. This is because only a tiny portion of far-red light is used in photosynthesis. On the other hand, Caddell et al. mentioned that antenna assembly component genes CpSRP43, CpSRP54a, and their paralog, CpSRP54b, have a high photosynthetic contribution to chlorophyll content. This impacts plants that grow in mixed communities.

The articles included in this Research Topic contribute to a better understanding of the many facets that light plays to enhance photosynthesis and improve plant growth under controlled conditions. There are still several aspects to be studied, such as the varietal response and the effects of light on plants exposed to nanoagrochemicals.
